# High prevalence of asymptomatic nosocomial candiduria due to *Candida glabrata* among hospitalized patients with heart failure: a matter of some concern?

**DOI:** 10.18502/cmm.6.4.5327

**Published:** 2020-12

**Authors:** Seyed Reza Aghili, Mahdi Abastabar, Ameneh Soleimani, Iman Haghani, Soheil Azizi

**Affiliations:** 1 Invasive Fungi Research Center, Communicable Diseases Institute, Mazandaran University of Medical Sciences, Sari, Iran; 2 Department of Medical Mycology, Faculty of Medicine, Mazandaran University of Medical Sciences, Sari, Iran; 3 Department of Laboratory Medicine, Faculty of Allied Medical Sciences, Mazandaran University of Medical Science, Sari, Iran

**Keywords:** *Candida glabrata*, Heart failure, Hospitalized patients, Nosocomial candiduria

## Abstract

**Background and Purpose:**

Heart failure is a leading cause of hospitalization, and asymptomatic candiduria is common in hospitalized patients with low morbidity. However, in most patients, it is resolved spontaneously on the removal of the catheter. Despite the publication of guidelines, there are still controversies over the diagnosis and management of candiduria. However, in hospitalized patients with heart failure, the decision to treat candiduria is especially important since the nosocomial infections are associated with an increase in morbidity, mortality, length of hospital stay, and healthcare costs. Some species of *Candida*, such as *Candida glabrata*, are increasingly resistant to the first-line and second-line antifungal medications. The present study aimed to investigate the incidence of asymptomatic *Candida* urinary tract infection due to *C. glabrata* and antifungal susceptibility of *Candida* isolates in hospitalized patients with heart failure.

**Materials and Methods:**

In total, 305 hospitalized patients with heart failure were studied to identify asymptomatic nosocomial candiduria during 2016-17 in one private hospital in the north of Iran. The Sabouraud’s dextrose agar culture plates with a colony count of >10^4^ colony-forming unit/ml of urine sample were considered as *Candida* urinary tract infection. *Candida* species were identified based on the morphology of CHROMagar* Candida* (manufactured by CHROMagar, France) and PCR-RFLP method with *Msp*I restriction enzyme. Antifungal susceptibility testing of the isolates was performed using five mediations, including itraconazole, voriconazole, fluconazole, amphotericin B, and caspofungin by broth microdilution method according to CLSI M27-S4.

**Results:**

In this study, the rate of asymptomatic *Candida* urinary tract infection was 18.8%, which was more common in people above 51 years old and females (70%). In addition to the urinary and intravascular catheter, the occurrence of candiduria in hospitalized patients had significant relationships with a history of surgical intervention, diastolic heart failure, and use of systemic antibiotics (P>0.05). Among* Candida* spp., non-*albicans Candida* species was the most common infectious agent (59.7%). Moreover, *C. glabrata* (n=27, 40.3%) (alone or with other species) and *Candida albicans* (n=27, 40.3%) were the most common agents isolated in *Candida* urinary tract infection. Based on the results of the in vitro susceptibility test, the *C. glabrata* isolates were 15%, 59%, 70%, 74%, and 85% susceptible to caspofungin, amphotericin B, itraconazole, voriconazole, and fluconazole, respectively.

**Conclusion:**

According to the findings, there was a high prevalence of asymptomatic *Candida* urinary tract infection in hospitalized patients with heart failure. Besides, it was suggested that there was a shift towards non-*albicans Candida*, especially *C. glabrata*, in these patients. Therefore, asymptomatic candiduria in hospitalized patients with heart failure should be considered significant. Furthermore, the identification of *Candida* species along with antifungal susceptibility is essential and helps the clinicians to select the appropriate antifungal agent for better management of such cases.

## Introduction

Heart failure (HF) is a leading cause of hospitalization among the elderly (i.e., people over 65 years old) in the world, and hospitalization is associated with substantial mortality rates. The HF and diabetes are two diseases associated with metabolic disorders since they lead to imbalance or abnormalities in biochemical and physiological factors of the human body [ 1 , 2 ]. Rate of co-existence of HF and diabetes has been reported to be as high as almost 40% [ 2 ]. Infections are the main co-morbidities diagnosed in hospitalized HF patients [ 3 ]. 

Alon et al. in their research project studied 9,335 HF patients and found that 3,530 (38%) of them were hospitalized at least once due to urinary tract infections (UTIs) which is one of the most frequent diagnoses in these patients (15.7%) [ 4 ]. The UTIs are caused by microbes, such as bacteria and fungi, and can affect the kidneys, bladder, and the tubes that run between them. The heart and small intestine are connected to urinary systems and the most relevant organs to UTIs [ 5 ]. Based on previous studies, this infection occurs more often in females, compared to males, with a ratio of 8:1 [ 6 ]. 

Moreover, the findings of previous studies have indicated that almost 50% of hospitalized patients with HF use urinary catheters due to urinary incontinence and overactive bladder [ 7 , 8 ]. Urinary catheterization increases the risk of nosocomial UTIs up to 97% [ 9 ] and the longer durations of catheter usage lead to the appearance of more infectious organisms in the urine [ 10 ]. Fungal UTIs caused by yeast, such as Candida species, have increased in hospitalized patients over the last decade, especially HF patients [ 11 ]. 

Candiduria (i.e., the presence of Candida yeasts in urine) is a marker of colonization or infection in the lower or upper urinary tract by Candida species. Patients with candiduria can be categorized as asymptomatic or symptomatic based on the diagnostic criteria and obtained data. Asymptomatic candiduria is defined as a positive urine culture with ≥ 10^3^ yeast colonies/ml in the absence of dysuria, polyuria, flank pain, and/or fever. Asymptomatic catheter-associated candiduria is common in hospitalized patients and has low morbidity. Moreover, it is resolved spontaneously by the removal of the catheter in most patients. Despite the publications of guidelines in this regard, there are controversies over the diagnosis and management of candiduria [ 12 , 13 ]. 

Moreover, candiduria may be a symptom of systemic candidiasis which is developed due to hematogenous seeding of yeast in patients. Clinical findings vary and often include asymptomatic or rarely symptomatic patients with cystitis, pyelonephritis, prostatitis, epididymo-orchitis, or urinary tract fungus balls. Despite the fact that *Candida albicans* species is the most frequently identified isolate of candiduria in hospitals, non-*albicans Candida* species now account for a significant proportion of clinical isolates collected worldwide in hospitals. Furthermore, they are also important due to the increasing resistance to antifungal agents [ 14 , 15 ]. 

Candiduria caused by *Candida glabrata* is now more common than that caused by other *Candida* species in some geographic areas and some patient groups [ 16 - 18 ]. The *C. glabrata *infections have a high mortality rate in immunocompromised hospitalized patients; therefore, it is essential to evaluate candidiasis in hospitalized patients with HF. Regarding the high resistance of some non-*albicans Candida* species, such as *C. glabrata* and *C. krusei*, to antifungal agents [ 19 , 20 ], the isolation and detection of species of the infecting agent in urine samples of HF patients should be considered important for the treatment. 

In this study, a laboratory-based survey was conducted using the CHROMagar *Candida* (CHROM agar, France) culture and polymerase chain reaction-restriction fragment length polymorphism (PCR-RFLP) method with *Msp*I restriction enzyme for the identification of candiduria agents. Moreover, antifungal susceptibility tests were performed on the isolates using the broth microdilution method and Clinical Laboratory Standards Institute (CLSI) documents with five medications to find the most appropriate treatment. Based on the results of the present study, *C. glabrata* is an emerging menace that leads to the development of candiduria in hospitalized patients with HF.

## Materials and Methods

This prospective, descriptive cross-sectional, laboratory-based surveillance study was carried out from July 2016 to December 2017 in a Private Heart Center in Sari, north of Iran. In total, 305 hospitalized patients with HF were investigated to identify asymptomatic candiduria and determine their etiologic agents. This research was approved by the Ethics Committee of Mazandaran University of Medical Sciences, Sari, Iran (ethics code: IR.MAZUMS.REC.96.3045). In this regard, informed consent was obtained from the patients and they were told that their participation was voluntary; accordingly, they were able to withdraw from the study at any stage without any consequences. 

According to the research objectives, the HF patients included in the study had no previous or current urinary tract infection based on the physical examination on the day of their admission to the hospital. Moreover, they had been using an indwelling urinary catheter for more than three days by the time of the study. The patients who did not use a urinary catheter, had been using a urinary catheter for less than three days, and had a previous or current urinary tract infection were excluded from the study. The required data were collected through questionnaires and included demographic characteristics (e.g., age and gender), underlying diseases (e.g., diabetes and a form of HF), and risk factors (e.g., long hospital stay, usage of several catheters, and treatment with antibiotics, corticosteroids, and antifungal medications).

In total, 580 urine samples were collected from 305 HF patients and transferred immediately to the hospital pathology lab. A urine wet mount examination was performed to check for pus cells, red blood cells, or any fungal elements. In total, 100 µl of each un-centrifuged urine sample was cultured after shaking two culture media. One sample was cultured on Sabouraud’s dextrose agar (SDA) (manufactured by Quelab, Canada) with chloramphenicol (100mg/L) and the other sample was cultured on brain heart infusion agar (BHI) (manufactured by Quelab) with chloramphenicol (100mg/L). 

All plates were incubated at 37 °C up to a maximum of one week. The Candida species that were cultivated on culture plates with a colony count of > 10^4^ colony-forming unit (CFU)/ml or 10^3^ < colony count < 10^4^ CFU/ml associated with pyuria in urine sample were considered significant. Subsequently, 5-10 ml of blood sample of patients with candiduria was collected by venipuncture aseptically and processed according to the standard protocols. 

### Isolates identification and antifungal susceptibility testing

**Figure 1:**
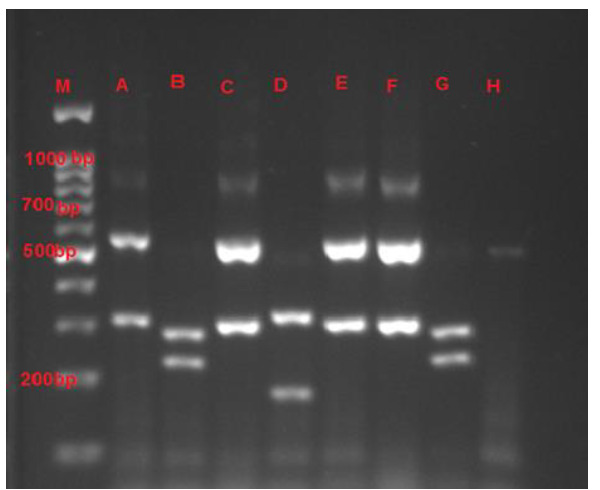
Agarose gel electrophoresis of digested ITS-PCR products of some isolates. M: molecular marker, A: Candida glabrata, B: Candida albicans, C: Candida glabrata, D: Candida tropicalis, E: Candida glabrata, F: Candida glabrata, G: Candida albicans, and H: Candida parapsilosis

The species were identified based on colony morphology on CHRO Magar *Candida* and the PCR-RFLP method [ 21 ]. The PCR-RFLP method was performed by genomic DNA extraction by the phenol-chloroform and amplification of yeast gene using the internal transcribed spacer (ITS) 1 (forward: 5´-TCCGTA-GGT-GAA-CCT-GCG-G-3´) and ITS4 (reverse: 5´-TCC-TCC-GCT-TATTGA-TAT-GC-3´) primers (manufactured by MWG-Biotech AG, Germany). Afterward, the *Msp*I restriction enzyme (manufactured by Thermo Fisher Scientific, USA) was used for the digestion of PCR products, and restriction fragments were separated using 2% agarose gel electrophoresis. Figure 1 shows the exact size of digested ITS-PCR products of some isolates.

The *Msp*I enzyme cannot cause cleavage in the ITS region of *C. parapsilosis*. Therefore, the PCR- Hyphal wall protein 1 (HWP1) discriminatory pattern was performed by the amplification of the HWP1 gene as described by Abastabar et al. [ 22 ] to distinguish *C. parapsilosis* and *C. orthopsilosis*. The PCR amplification of the HWP1 gene for *C. parapsilosis* was achieved using the forward, 5′-CGAGG TGAATATGATGCTTGTA-3′ and reverse, 5′-CCAACAGAATTGCTTAATACCATA-3′, for C. orthopsilosis forward, 5′-ACCACCACCTAGTTCTGAG-3′ and reverse, and 5′-TCACTTGGAAGATTGAGAATAACA-3′ primer pairs. They produce two different DNA fragments which are approximately 840 and 900 bp for *C. parapsilosis* and *C. orthopsilosis*, respectively.

For *Candida* species isolates, in-vitro antifungal susceptibility testing was performed using broth microdilution and CLSI document M27-S4 (Reference Method for Broth Dilution Antifungal Susceptibility Testing of Yeasts, Approved Standard, CLSI, Wayne, PA, USA, 2012.) [ 23 ]. All isolated *Candida* species were tested by fluconazole (FLC) and voriconazole (VRC) (Pfizer, Sandwich, United Kingdom), itraconazole (ITC) (manufactured by Janssen, Belgium), caspofungin (CAS) (manufactured by Merck Sharp & Dohme B.V.), and amphotericin B (AMB) (manufactured by Bristol-Myers-Squibb, The Netherlands). The *C. parapsilosis* (ATCC 22019) and C. krusei (ATCC 6258) strains were used as the controls.

Powders of ITC, VRC, AMB, CAS, and FLC antifungal agents were obtained from the manufacturers. Final concentrations of antifungal agents in the wells were within the ranges of 0.016-16 μg/ml for ITC, VRC, and AMB, 0.008-8 μg/ml for CAS, and 0.064-64 μg/ml for FLC. Stock solutions of medications were prepared in dimethyl sulfoxide except for CAS and FLC which were dissolved in sterile water and stored at –80 °C until they were used. 

The isolated Candida species were grown on SDA and incubated at 35 °C for 48 h. A spectrophotometer at 530 nm was used to adjust a conidial inoculum with a range of 1-5⨯10^5^ CFU/ml by suspensions diluted in RPMI 1640 medium. The medication containing 96-well plastic micro-plates was inoculated with this suspension and incubated at 35 °C for 24–48 h. The minimum inhibitory concentrations (MICs) of FLC, VRC, CAS, ITC, and amphotericin B were determined according to the CLSI M27-S4 guidelines. Resistance breakpoints for *Candida* species to the different antifungal medications were also selected based on CLSI M27-S4 guidelines [ 24 ]. 

### Statistical analysis

The collected data were analyzed in SPSS software (version 19) and the quantitative variables were described using mean and standard deviation. Moreover, the percentage and frequency were calculated for qualitative variables. The Chi-square test and t-test were used to determine differences between experimental factors and the association of the groups with each other. In addition, non-parametric tests were used to compare the two groups. It should be noted that a p-value of less than 0.05 was considered statistically significant. 

## Results

**Figure 2:**
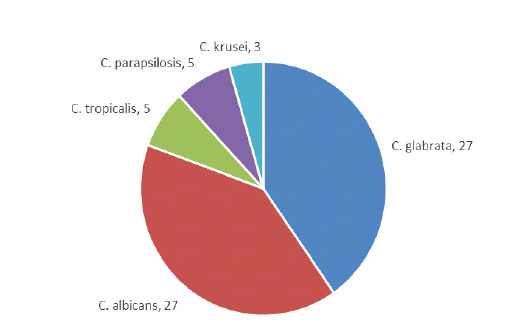
Frequency of Candida species isolated from heart disorder patients with asymptomatic candiduria

According to the mycology laboratory results, out of the 305 hospitalized patients who met the inclusion criteria, 58 (18.8%) cases had asymptomatic *Candida* urinary tract infection (*Candida* colony count: >10^4^ CFU/ml or 10^3^<colony count<10^4^ CFU/ml associated with pyuria). The *C. glabrata* (n=27, 40.3%) and *C. albicans* (n=27, 40.3%) were the most common agents isolated from candiduria-infected patients. Frequency of isolated *Candida* species from HF patients with asymptomatic candiduria is presented in Figure 2.

**Figure 3:**
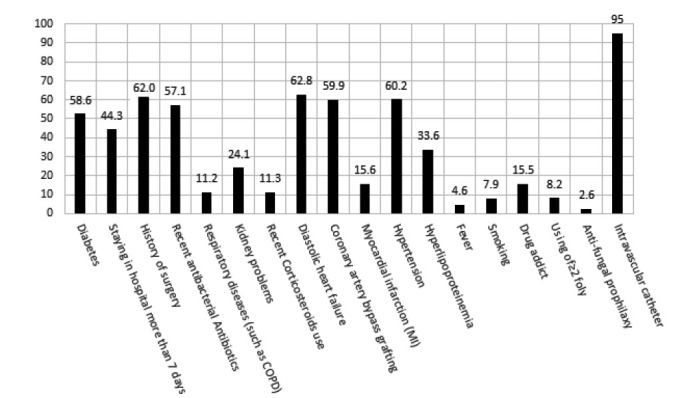
Percentage frequency of underlying condition in patients with a heart disorder

No positive blood culture was found in patients with candiduria. The clinical information and demographic features of 305 patients were collected for the purposes of the study. In total, 183 (57.9%) of subjects were female and 122 (42.1%) of them were male. The patients were within the age range of 31-88 years and their mean age was 67.6 years. Based on the results, female subjects were at higher risk of heart disease, compared to males; however, this difference was not significant (P>0.05). Prevalence of heart disorders was different in various age groups and peaked at age ranges of 51-65 and 66-80 years in both males and females. Intravascular catheter insertion (95%), diastolic HF (62.8%), history of surgery (62.0%), coronary artery bypass grafting (59.9%), diabetes mellitus (58.6%), use of broad-spectrum antibacterial antibiotics (57.1%), and hospitalization for seven days or more (44.3%) were the major underlying conditions in these patients (Figure 3).

Based on their medical records, 159 (59.1%) cases had diabetes as an added underlying condition. Moreover, heart disorders were associated with diabetes in 60.7% and 39.3% of female and male patients, respectively. However, diabetes caused no statistically significant difference between patients with and without candiduria (P> 0.05). 

The most common underlying conditions significantly associated with candiduria in hospitalized patients with HF were a history of surgical intervention, diastolic HF, and systemic antibiotics therapy (P<0.05). 

The *C. albicans* (40% alone and 6.9% co-isolated with *C. glabrata*) and *C. glabrata* (31% alone and 15.5% co-isolated with other species) had the most prevalence as candiduria agents ( Table 1). In total, nine patients had mixed infection caused by two different *Candida* species. All the mixed infection cases were caused by *C. glabrata* mixed with other species (four cases of *C. glabrata*+*C.* albicans, two cases of *C. glabrata*+*C.* tropicalis, two cases of *C. glabrata*+*C.* parapsilosis, and one case of *C. glabrata*+*C.* krusei). 

**Table 1:** Distribution of Candida spp. among heart failure patients with candiduria

*Candida* spp.	Frequency number (%)		
	Male	Female	Total
*Candida glabrata*	5 (28)	13 (33)	18 (31)
*Candida albicans*	6 (33)	17 (43)	23 (40)
*Candida tropicalis*	2 (11)	1 (2)	3 (5)
*Candida krusei*	1 (6)	1 (2)	2 (3)
*Candida parapsilosis*	0 (0)	3 (8)	3 (5)
*Mixed infection*	4 (22)	5 (12)	9 (16)
Total	18 (100)	40 (100)	58 (100)

### Susceptibility test results

The recent CLSI clinical M27-S4 approved breakpoint values were used for identification of the susceptibility of *Candida* species to antifungal medications [ 25 ]. Table 2 summarizes the main points of in vitro activity of five antifungal medications against all 67 isolates of *Candida* species. The FLC (0.25-64 μg/ml) showed the widest MIC range for all candidate species while VRC and AMB had the narrowest MIC range (0.016–16 μg/ml). The CAS and VRC had the lowest geometric mean MIC values against *C. albicans* (0.65 µg/mL) while *C. glabrata* (0.44 µg/mL) had the lowest geometric mean MIC value against VRC.

Among the isolated *Candida* species, *C. albicans* showed the greatest sensitivity to AMB (n=22; 81.5%) and CAS (n=15; 63.6%), in that order. Furthermore, the *C. glabrata* showed the greatest resistance and sensitivity to CAS (n=23; 85.2%) and VRC, in that order. All of the isolated *C. krusei* were resistant to ITC and FLU. The AMB was the most active medication particularly against *C. tropicalis* and *C. parapsilosis*. ITC, while FLU and VRC showed less activity against C. albicans, *C. krusei*, and *C. tropicalis*, in that order. However, in this study, ITC, VRC, and FLU had the most activity against C. glabrata isolates and more isolates in this species showed resistance to CAS and AMB.

**Table 2:** Activities of five antifungal medications against clinical isolates of five Candida species

Antifungal agent	Isolated Candida species	No. isolated	MIC 50 (µg/ml)	MIC 90 (µg/ml)	MIC Range	Geometric Mean	MIC Breakpoint M27S4 R (µg/ml)	Resistant No. (%)
Amphotericin B	*C. albicans*	27	2	4	0.016-8	1.43	>2	5 (18.5%)
	*C. glabrata*	27	2	16	0.25-16	2.40	>2	11 (40.7%)
	*C. tropicalis*	5	0.5	ND	0.5-2	ND	>2	0 (0%)
	*C. parapsilosis*	5	1	ND	1-2	ND	>2	0 (0%)
	*C. krusei*	3	2	ND	2-8	ND	>2	1 (33.3%)
Caspofungin	*C. albicans*	27	0.5	4	0.032-16	0.65	≥1	12 (44.4%)
	*C. glabrata*	27	1	4	0.032-8	1.08	≥0.5	23 (85.2%)
	*C. tropicalis*	5	2	ND	0.125-8	ND	≥1	3 (100%)
	*C. parapsilosis*	5	1	ND	0.25-8	ND	≥8	2 (40%)
	*C. krusei*	3	2	ND	1-4	ND	≥1	2 (66.8%)
Itraconazole	*C. albicans*	27	16	16	0.032-16	4.32	≥1	20 (74.1%)
	*C. glabrata*	27	2	16	0.5-16	2.28	≥1	8 (29.6%)
	*C. tropicalis*	5	1	ND	1-16	ND	≥1	2 (40%)
	*C. parapsilosis*	5	1	ND	0.5-1	ND	≥1	5 (100%)
	*C. krusei*	3	16	ND	2-16	ND	≥1	3 (100%)
Voriconazole	*C. albicans*	27	16	16	0.016-16	2.22	≥1	19 (70.4%)
	*C. glabrata*	27	0.125	16	0.0625-16	0.44	≥1	7 (25.9%)
	*C. tropicalis*	5	0.5	ND	0.0625-16	ND	≥1	2 (40%)
	*C. parapsilosis*	5	0.0625	ND	0.032- 0.125	ND	≥1	0 (0%)
	*C. krusei*	3	16	ND	0.5-16	ND	≥2	2 (66.8%)
Fluconazole	*C. albicans*	27	64	64	0.25-64	14.81	≥8	19 (70.4%)
	*C. glabrata*	27	8	64	2-64	11.46	≥64	7 (14.8%)
	*C. tropicalis*	5	4	ND	2-64	ND	≥8	2 (40%)
	*C. parapsilosis*	5	4	ND	2-16	ND	≥8	1 (20%)
	*C. krusei*	3	64	ND	64	ND	≥8	3 (100%)

MIC: minimum inhibitory concentration>

## Discussion

The *C. albicans* was the most important yeast associated with human candiduria in the last decades. Reported incidence of candiduria varies (10-30%) in different geographical locations [ 26 - 29 ]. In addition, this rate has increased due to the use of broad-spectrum antibiotics [ 30 ] or other underlying conditions, such as old age [ 31 ], HF disease [ 32 ], and long hospital stay [ 33 ].

The *C. glabrata* is a non-pathogenic normal flora of healthy individuals, and it is rarely associated with candiduria in hospitalized patients [ 34 ]. However, it is now the second or third most frequently isolated *Candida* species from *Candida* urinary tract infection [ 35 - 37 ]. In the last two decades, an important shift was observed in nosocomial *Candida* infections regarding the type of *Candida* species from *C. albicans* to more treatment-resistant non-albicans species [ 38 , 39 ]. Moreover, according to previous studies, the prevalence of candiduria caused by *C. glabrata* has increased in the last two decades [ 40 - 42 ] which has raised concerns in the medical mycology due to the organisms therapeutic problems and resistance to common antifungal medications. 

In the present study, it was found that similar to *C. albicans, C. glabrata* was associated with candiduria in HF patients. Moreover, the mixed growth of *C. glabrata* and other *Candida* species (i.e., *C. albicans*, *C. tropicalis*, *C. parapsilosis*, and *C. krusei*) were observed in nine patients. Based on the results of some studies , *C. glabrata* establishes competitive interactions with other species during biofilm formation and candidiasis development [ 43 , 44 ]. According to the findings of other studies in Iran, *Candida albicans* is the most common isolated species from candiduric patients (50–70%). However, recently, due to the increasing resistance to antifungal medications, non-*albicans Candida* species, including, *C. glabrata* (almost 20%), *C. krusei*, *C. parapsilosis*, and *C. tropicalis* have also been implicated [ 45 - 47 ].

Occurrence of candiduria in hospitalized HF patients, even asymptomatic forms, increases the length of hospital stay and economic costs and contributes to antifungal overuse. Candiduria should be followed up among these patients since it can lead to some invasive candidiasis in them [ 48 ]. Catheters are the most common medical devices and almost two billion bladder catheters are inserted annually in the world [ 49 ]. In order to monitor urine output, it is common to use urinary catheterization in hospitalized patients with HF. However, the risk of urinary complications increases in long-term usage [ 50 ]. 

It is worth mentioning that the rate of catheter-associated infection was 10-30% overall [ 51 ]. Findings of the present study indicated that HF was more common in females, compared to men; nevertheless, the difference was not significant. According to previous studies, the incidence rate of HF has been rising faster in females (9%), compared to males (6%) during the past 20 years [ 52 ]. However, Ho et al. in a study performed in Framingham, USA found that the incidence of HF was significantly higher in males, compared to females in all age ranges [ 53 ].

Despite the fact that HF occurs in all ages, Health Harvard Publication reported that in the USA, the first heart attack usually occurs in people above 65 years old and it is the leading cause of death [ 54 ]. Findings of various research, including the present study, have indicated that intravascular catheter insertion [ 55 ], diastolic HF [ 56 ], history of surgery [ 57 ] particularly coronary artery bypass grafting [ 58 ], use of broad-spectrum antibacterial antibiotics [ 59 ], and staying in hospital for seven days or more [ 46 ] are major risk factors for candiduria in HF patients which are also statistically significant. 

In this study, diabetes was a co-morbidity for over half of HF patients (58.6%); however, there was no significant difference in this regard between the patients with candiduria and patients without candiduria. Therefore, diabetes is a risk factor for HF patients, and the problems due to HF are considered as predisposing factors for opportunistic infection, such as candidiasis [ 59 ]. Many types of medical devices have been used for the treatment of HF patients which can lead to a potential risk of the adhesion of Candida species and cause biofilm-associated candiduria. In recent years, several studies conducted in Iran [ 46 , 60 , 61 ] and around the world [ 62 ] have focused on the role of non‐*albicans Candida* species, especially *C. glabrata*, in candiduria.

In this study, CAS and AMB were the most active antifungals for both *C. albicans* and *C. glabrata* isolates. Shokohi et al. [ 63 ] reported 98% and 99.5% susceptibility to CAS and AMB in *C. albicans* isolated from cancer patients in the north of Iran. 

The *C. albicans* strains were 74.1%, 70.4%, and 70.4% resistant to ITC, VRC, and FLU, respectively, while, *C. glabrata* isolates were 29.6%, 25.9%, and 14.8% resistant to ITC, VRC, and FLU, respectively. Aslani et al. have also reported that *C. albicans* isolates are highly resistant to ITC, VRC, and FLU [ 64 ]. According to the findings of previous studies, resistance to azoles in *C. glabrata* has a multifactorial nature [ 65 ]. Based on the results of a study conducted by Amirrajab N. et al. [ 66 ], the overall rates of resistance to ITC, VRC, and FLU were 72.5%, 47.5%, and 10% respectively, which are inconsistent with those of the present study.

Incipient menace of non-*albicans* species other than *C. albicans* and mixed infections, such as candiduria, in hospitalized patients with HF, indicates that the epidemiology of candiduria agents is changing. These types of infections may need higher doses or special antifungal agents and may be resistant to common treatment. 

Therefore, treatment of candiduria in these patients with FLU, CAS, and AMB may be inefficient. Improved diagnosis methods, such as culture on to chromogenic media and PCR-RFLP molecular detection, have shown that other *candida* species are important pathogens [ 67 ]. 

## Conclusion

Based on the findings of this study, there was a high prevalence of asymptomatic nosocomial candiduria in hospitalized patients with HF. It is suggested to shift towards non-*albicans Candida*, especially *C. glabrata* in these hospitalized patients. Candiduria may be the first symptom of disseminated candidiasis with high morbidity and mortality, especially in the presence of risk factors, such as immunosuppression, catheterization, cardiovascular disease, prolonged hospital stay, and use of antibacterial antibiotics. Recognition and management of these patients are difficult for clinicians since it has no typical symptoms. Susceptibility of *C. glabrata* to antifungal agents is different from *C. albicans* and other *candida* species; therefore, asymptomatic nosocomial candiduria in HF patients should be considered. Identification of *Candida* species along with antifungal susceptibility is important and helps the clinicians to select the appropriate antifungal agent for better management of such cases.

## Authors’ contribution

SR. A. conceived the study. A. S. and S. A. prepared the strains. A. S. and I. H. performed the experiments. SA. A. and M. A prepared the manuscript. All authors read and approved the final manuscript.

## Financial disclosure

No financial interests related to the material of this manuscript have been declared.
